# Disease Progression in *CNGA3* and *CNGB3* Retinopathy; Characteristics of Slovenian Cohort and Proposed OCT Staging Based on Pooled Data from 126 Patients from 7 Studies

**DOI:** 10.3390/cimb43020067

**Published:** 2021-08-16

**Authors:** Manca Tekavčič Pompe, Nika Vrabič, Marija Volk, Andrej Meglič, Martina Jarc-Vidmar, Borut Peterlin, Marko Hawlina, Ana Fakin

**Affiliations:** 1 Eye Hospital, University Medical Centre Ljubljana, 1000 Ljubljana, Slovenia; manca.tekavcic-pompe@guest.arnes.si (M.T.P.); vrabicnika@gmail.com (N.V.); andrej.meglic.info@gmail.com (A.M.); martina.jarcvidmar@gmail.com (M.J.-V.); marko.hawlina@gmail.com (M.H.); 2 Clinical Institute of Genomic Medicine, University Medical Centre Ljubljana, 1000 Ljubljana, Slovenia; marija.volk@kclj.si (M.V.); borut.peterlin@guest.arnes.si (B.P.)

**Keywords:** *CNGA3*, *CNGB3*, achromatopsia, cone-dystrophy, nystagmus, foveal hypoplasia, color vision, autofluorescence, FAF, optical coherence tomography, OCT, electroretinography, ERG, disease stage, progression, degeneration

## Abstract

Achromatopsia has been proposed to be a morphologically predominately stable retinopathy with rare reports of progression of structural changes in the macula. A five-grade system of optical coherence tomography (OCT) features has been used for the classification of structural macular changes. However, their association with age remains questionable. We characterized the Slovenian cohort of 12 patients with pathogenic variants in *CNGA3* or *CNGB3* who had been followed up with OCT for up to 9 years. Based on observed structural changes in association with age, the following four-stage classification of retinal morphological changes was proposed: (I) preserved inner segment ellipsoid band (Ise), (II) disrupted ISe, (III) ISe loss and (IV) ISe and RPE loss. Data from six previously published studies reporting OCT morphology in *CNGA3* and *CNGB3* patients were additionally collected, forming the largest *CNGA3/CNGB3* cohort to date, comprising 126 patients aged 1–71 years. Multiple regression analysis showed a significant correlation of OCT stage with age (*p* < 0.001) and no correlation with gene (*p* > 0.05). The median ages of patients with stages I–IV were 12 years, 23 years, 27 years and 48 years, respectively, and no patient older than 50 years had continuous ISe. Our findings suggest that achromatopsia presents with slowly but steadily progressive structural changes of the macular outer retinal layers. However, whether morphological changes in time follow the proposed four-stage linear pattern needs to be confirmed in a long-term study.

## 1. Introduction

Achromatopsia (ACHM) is a complex inherited retinal disorder primarily affecting the cone cell function [1], the incidence being around 1:30,000 births. The main clinical features are affected color vision, nystagmus, light sensitivity and often poor visual acuity [2,3].

The mode of ACHM inheritance is autosomal recessive with currently six known causative genes: *CNGA3* [4], *CNGB3* [5], *GNAT2* [6], *PDE6C* [7], *PDE6H* [8] and *ATF6* [9]. The majority of causative genes (with the exception of ATF6) are involved in the cone-specific phototransduction cascade. *CNGA3* and *CNGB3* genes encode the alpha and beta subunits of the cyclic nucleotide-gated ion channels, respectively, located in the plasma membrane of outer cone segments [10,11]. Cumulatively, there are over 150 known pathogenic variants in both *CNGA3* and *CNGB3* genes, accounting for over 70% of all ACHM cases [1].

ACHM has been described as a clinically heterogeneous disease with significant variability of symptoms among patients [12]. The “Complete” form of ACHM, also known as typical ACHM or rod monochromatism; and “incomplete” form of ACHM have been described, the latter characterized by better visual acuity and usually some residual color discrimination [1].

Workup of ACHM patients includes slit-lamp exam, visual electrodiagnostic testing (focusing on electroretinogram (ERG) and visual evoked potentials (VEP)), optical coherence tomography (OCT), fundus autofluorescence (FAF), color vision tests and perimetry (VF) [13]. Fundus appearance of patients with ACHM is usually normal; sometimes, an absent foveal reflex or slight mottling of the macular pigment is seen [14]. Electrophysiological measurements typically show reduced or absent cone responses, whereas rod responses remain normal or near-normal [15]. In some patients, deficits in rod-mediated and rod-cone-mediated functions have also been demonstrated [16,17]. OCT of the macula shows either continuous inner segment ellipsoid (ISe) or disrupted or absent ISe. In some patients, an optical gap, also termed hyporeflective zone or bubble, is observed in the fovea [12,18,19,20]. FAF can be normal or show hypo- or hyper-autofluorescence and in some cases, a hypoautofluorescent ring in the foveal region [21,22,23]. CVT show markedly reduced or even absent color discrimination [24]. VF are often difficult to test objectively in ACHM patients due to nystagmus and unsteady fixation; however, central scotoma can be detected [2].

Several studies explored the clinical course of ACHM patients to assess disease progression [18,19,22,25,26,27,28]. Although some reports showed visual and structural deterioration [18,19,22,28,29], other studies report no correlation of clinical presentation with age [12,17,25]. Consensus on the matter has been difficult to reach due to the lack of large cross-sectional studies and/or longitudinal studies spanning several decades.

The purpose of this study is to characterize the Slovenian cohort with pathogenic variants in *CNGA3* and *CNGB3* with a focus on OCT changes and to perform a cross-sectional analysis of pooled OCT data from ours and previously published studies to determine whether there is any age-related structural degeneration in the macula with age.

## 2. Materials and Methods

### 2.1. Patients

The study included 12 patients from 9 families recruited from the Eye Hospital, University Medical Centre Ljubljana (UMC LJ), Slovenia, who had been referred for genetic testing due to suspected inherited retinal disease (IRD) and had confirmed biallelic pathogenic or likely pathogenic variants in *CNGA3* (*n* = 7) or *CNGB3* (*n* = 5). The included cohort consisted of 5 male and 7 female patients. Their median age at the first exam was 7 months (range, 3 months–70 years). The duration of the follow-up period varied among patients; 11 patients had follow-up of median 5 years (range 5 months to 31 years). The median age for the last exam was 8 years (range, 1–71). One patient presented to our outpatient clinic only once so far with no follow-up data available. Although other ACHM-related genes are also routinely screened in patients with suspected IRDs, no other ACHM-related genes were identified in patients from UMC LJ, which is the only tertiary center overseeing IRD patients in a country of 2 million people.

The study was conducted in agreement with the Declaration of Helsinki. Informed written consent was obtained from the patients.

### 2.2. Genetic and Bioinformatic Analysis

Sequencing of the defined clinical target was performed using next-generation sequencing on the isolated DNA sample. The fragmentation and enrichment of the isolated DNA sample were performed according to the protocol Twist CORE Exome or Nextera Coding Exome, with subsequent sequencing on Illumina NovaSeq 6000 (Cegat, Tübingen, Germany) or Illumina NextSeq 550 (UMCL, Ljubljana, Slovenia) in 2 × 150 cycles or 2 × 100 cycles, respectively. After duplicates were removed, the alignment of reads to UCSC hg19 reference assembly was done using the BWA algorithm (v0.6.3), and variant calling was done using the GATK framework (v2.8). Only variants exceeding the quality score of 30.0 and depth of 5 were used for downstream analyses. Variant annotation was performed using ANNOVAR and snpEff algorithms, with pathogenicity predictions in dbNSFPv2 database. Reference gene models and transcript sequences are based on RefSeq database. Structural variants were assessed using CONIFER v0.2.2 algorithm. Variants with population frequency exceeding 1% in gnomAD (gnomad.broadinstitute.org), synonymous variants, intronic variants and variants outside the clinical target were filtered out during analyses. The bioinformatics analysis was performed according to the modified analytical pathway of the Center for Mendelian Genomics, (version 0.5). The interpretation of sequence variants was based on ACMG/AMP standards and guidelines [30].

In family cases, segregation analyses were performed in siblings, using Sanger sequencing for targeted known pathogenic variants (patient’s IDs: 6, 10 and 12). In one case (patient #9), whole-genome sequencing was used to detect duplication of exon 7 in the *CNGA3* gene.

### 2.3. Clinical Examination 

Patients underwent a complete ophthalmic examination, which included best-corrected visual acuity (BCVA), color vision testing, slit-lamp biomicroscopy, dilated fundus examination, imaging and visual electrodiagnostics.

#### 2.3.1. Visual Function Tests 

BCVA was determined using the age-appropriate test: Snellen chart, Lea symbols chart or preferential looking test. For statistical purposes, the visual acuity of the better eye (or binocular in small children) was expressed in the decimal scale and converted into logMAR. Color vision was tested using Ishihara plates.

#### 2.3.2. Imaging

FAF of the macula (30°) was performed using Heidelberg Spectralis (Spectralis; Heidelberg Engineering, Heidelberg, Germany). External fixation light was used to acquire FAF of the peripheral retina in one patient (patient #3). Optical coherence tomography (OCT) extending 8 mm of the macula was performed using Spectral-domain OCT (SD-OCT; Heidelberg Spectralis) or Swept-source OCT (SS-OCT; Triton^TM^, Topcon, Tokyo, Japan). The integrity of hyperreflective bands on OCT, representing ISe, external limiting membrane (ELM) and RPE, was determined qualitatively on OCT scan through the fovea. Each OCT figure was graded into one of five grades proposed by Aboshiha et al. [22]. Foveal hypoplasia was graded according to Thomas et al. [31]. Central retinal thickness in the region extending 1 mm centred in the fovea (CRT) was measured automatically with Spectralis or Triton software, respectively.

#### 2.3.3. Visual Electrodiagnostics

In 7 out of 12 cases, ERG recordings were first performed in the early infancy (median age 7 months, range 3–12 months). Because proper dark adaptation can be difficult in infants and small children, ERG recordings were done according to GOSH [32]. Skin electrodes (Natus neurology, Middleton, WI, USA) were applied just below the lower lids. Scotopic ERGs were recorded to white scotopic and blue flashes, and photopic ERGs were recorded to white photopic (in a bright lit room) and 30 Hz flashes. Handheld Grass PS33 stimulator was used in combination with xenon light and color filters (Grass Technologies, West Warwick, Rhode Island, USA). Experiment control and signal acquisition were performed with Espion visual electrophysiology testing system (Diagnosys LLC, Littleton, MA, USA). Additional diagnostic insight was achieved by combining ERG measurements with VEP to exclude other pathologies. In adults, full-field ERG recordings were performed according to ISCEV standards [33]. Briefly, the recording HK-loop electrode [34] was hooked to the lower lid, and the reference and ground silver cup electrodes (Grass Technologies, West Warwick, RI, USA) were pasted at the level of the external canthus and on the forehead, respectively. The pupils were dilated with 1% tropicamide (Unimed Pharma, Bratislava, Slovakia). Scotopic and photopic ERGs were elicited with a full-field ColorDome stimulator (Diagnosys LLC, Littleton, MA, USA). Five different protocols were used: dark-adapted 0.01, 3.0 (flash intensity in cd·s·m−2) ERG, oscillatory potentials and light-adapted 3.0 and 30 Hz ERG. Children up to 7 years were recorded with skin instead of HK electrodes (Natus neurology, Middleton, WI, USA) without pupil dilation. Rod system function was assessed with dark-adapted 0.01 ERG (ISCEV) or dim blue flash (GOSH) protocols. Cone system function was tested with light-adapted 3.0 and 30 Hz flicker ERG or with white photopic and 30-Hz flashes. Due to high accuracy and agreement between a pediatric GOSH and the standard ISCEV protocol [35], results gained from both protocols were combined. 

### 2.4. Analysis of Previously Published Data 

Data was gathered from six previous studies reporting OCT features in *CNGA3* and *CNGB3* retinopathy [14,17,19,20,25,36]. Extracted data consisted of patients’ ID, age, gene, OCT grade and CRT. If OCT grading was stated for both eyes, the right eye was used. For patients with multiple visits, the first was used. If grading was not reported but images were shown, the grading was performed based on available images. Only patients with variants in a single gene (either *CNGA3* or *CNGB3*) were included. Only CRT measured with Spectralis were used for consistency. Multiple regression analysis was performed to determine whether there was any correlation between OCT features and age or gene.

## 3. Results

### 3.1. Genetic Findings

Genetic findings are presented in Table 1 and Table 2. The most prevalent pathogenic variants were CNGA3:c.847C>T (p.Arg283Trp) (seven alleles), followed by CNGA3:c.1641C>A (p.Phe547Leu) (four alleles) and *CNGB3*:c.1148delC (p.Thr383IlefsTer13) (four alleles) (Table 1). Other variants were presented in two alleles or only once. There was one novel variant, *CNGB3*:c.2104-2A>G, which presumably results in a null variant (intronic within ±2 of splice site). A copy number variation (CNV) was also identified by whole-genome sequencing—a CNV analysis using Delly software detected a duplication of exon 7 in the *CNGB3* gene, whereas the other variant in this patient was detected by exome sequencing.

### 3.2. Clinical Presentation

The patients’ clinical characteristics are summarized in Table 1.

#### 3.2.1. Visual Function

At their last exam at the median age of 8 years, the median BCVA on the better eyes was 0.16 (range, 0.01–0.6). Sixty-seven percent (8/12) of patients had visual acuity ≥ 0.05. There was no significant correlation between visual acuity and age (Pearson correlation, *p* > 0.05). There was no significant difference in the median visual acuity between *CNGA3* and *CNGB3* patients (Mann–Whitney U test, *p* > 0.05). 

**Figure 1:**
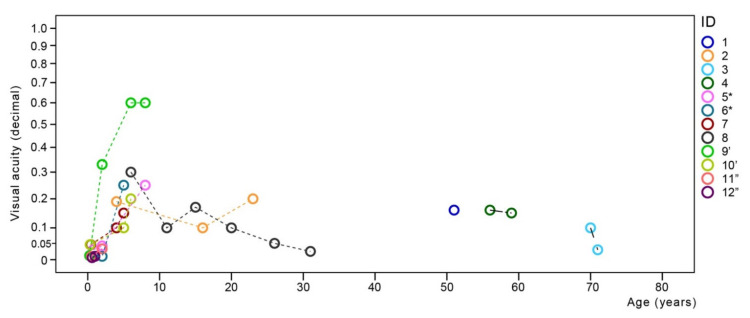
Visual acuity in relation to age. Chart showing best-corrected visual acuity in relation to age. Follow-up data are shown with interpolation lines. Note the increase of visual acuity in the first decade and relatively stable visual acuity afterwards with most patients retaining visual acuity of at least 0.05 decimal. Patients from the same families are marked with *, ’ and ”, respectively.

Longitudinal follow-up showed an initial increase in VA in the first decade (Figure 1). Color vision was measured in four patients. Three (*CNGA3*, ages 23–71 years) were only able to see the first Ishihara plate. Patient #8 (*CNGB3*, age 31 years) was able to discern 2/15 and 3/15 plates with the right and left eye, respectively. The visual perception of patient #8 was demonstrated by the patient using Adobe Photoshop (Adobe, San Jose, California) by modifying the red green and blue (RGB) color channels on the monochromatic image until it matched her perception of the source image (Figure S1).

#### 3.2.2. Optical Coherence Tomography and Fundus Autofluorescence Imaging

**Figure 2:**
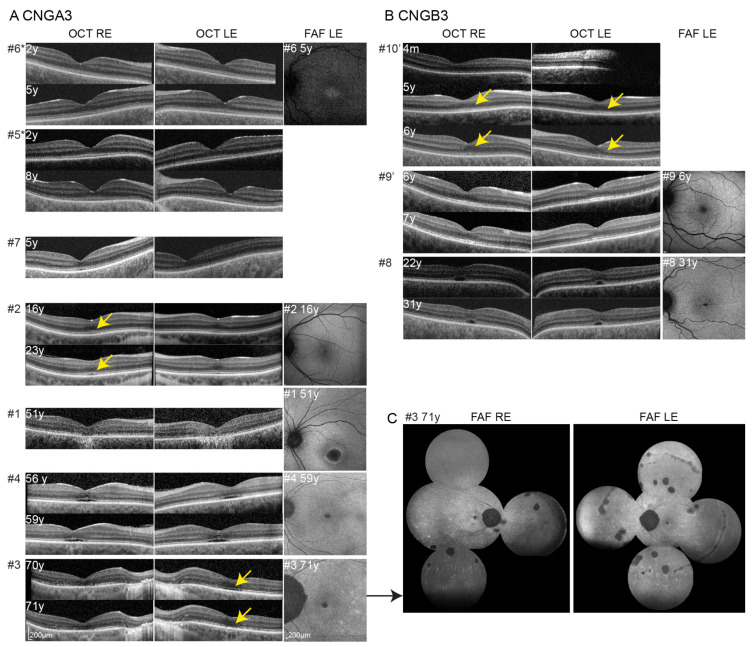
Optical coherence tomography and fundus autofluorescence. OCT images at first and follow-up imaging are shown for *CNGA3* (**A**) and *CNGB3* (**B**) patients. Patients are sorted from the youngest to the oldest from top to bottom. Patients’ number is stated with # on the left of each image. Changes noted on follow-up are marked with yellow arrows. FAF image is shown next to OCT, if available. FAF of the peripheral retina of patient #3 is shown in (**C**). Patients from the same families are marked with * and ’, respectively.

OCT was successfully performed in 10 patients (in 8 with a wide follow-up range of 1–9 years) and FAF in 7 patients (in 3 with follow-up of 1–3 years). Images are shown in Figure 2. Foveal hypoplasia was demonstrated in 70% (7/10) of imaged patients. All patients had mild (grade 1) foveal hypoplasia, five of them 1a and two 1b. There was no change in foveal hypoplasia grade on follow-up. 

AF showed increased signal in the youngest patient with the available image (age 5 years), a decreased signal in two patients (ages 6 years and 16 years) and a decreased signal with hyperautofluorescent border in the remaining four patients (ages 31–71 years). The oldest patient (#3, 71 years) who also underwent peripheral FAF imaging displayed scattered hyperautofluorescent dots and several areas of chorioretinal atrophy in the retinal periphery.

**Figure 3:**
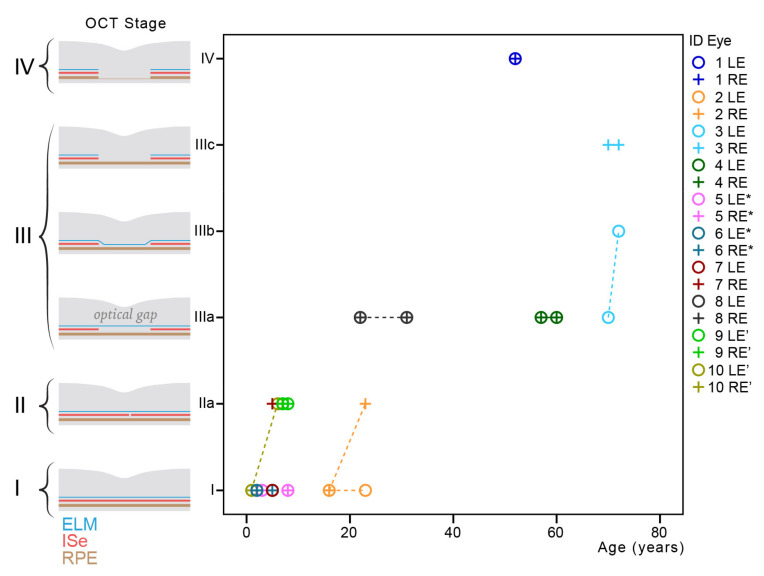
OCT features in relation to age. Chart showing OCT features in relation to age. Follow-up data are shown with interpolation lines. Note also good inter-eye symmetry. Patients from the same families are marked with * and ’ respectively.

The grading of photoreceptor structure on OCT according to [22] is stated in Table 2. At the first OCT imaging (at the median age of 11 years; range, 1–70 years), 45% of (9/20) eyes exhibited grade 1, and 65% (13/20) eyes exhibited grades 1–3. Stage 5 with RPE loss was present only in patient #1. There was good interocular symmetry with only two patients having different grades on right and left eyes. Only four eyes of three out of ten patients progressed in OCT grade on follow-up. Changes on follow-up are marked with yellow arrows in Figure 2. The right eye of patient #2 and both eyes from patient #10 progressed from grade 1 (normal) to grade 2 (disrupted ISe) in the age ranges of 16–23 and 5–6 years, respectively. The left eye of patient #3 changed from grade 4 (optical gap) to grade 3 (ISe loss) between ages 70 and 71 years. Considering only right eyes from the last exam, there was a significant correlation of grade with age (Pearson correlation, r = 0.75; *p* < 0.05). OCT features in relation to age are shown in Figure 3.

**Figure 4:**
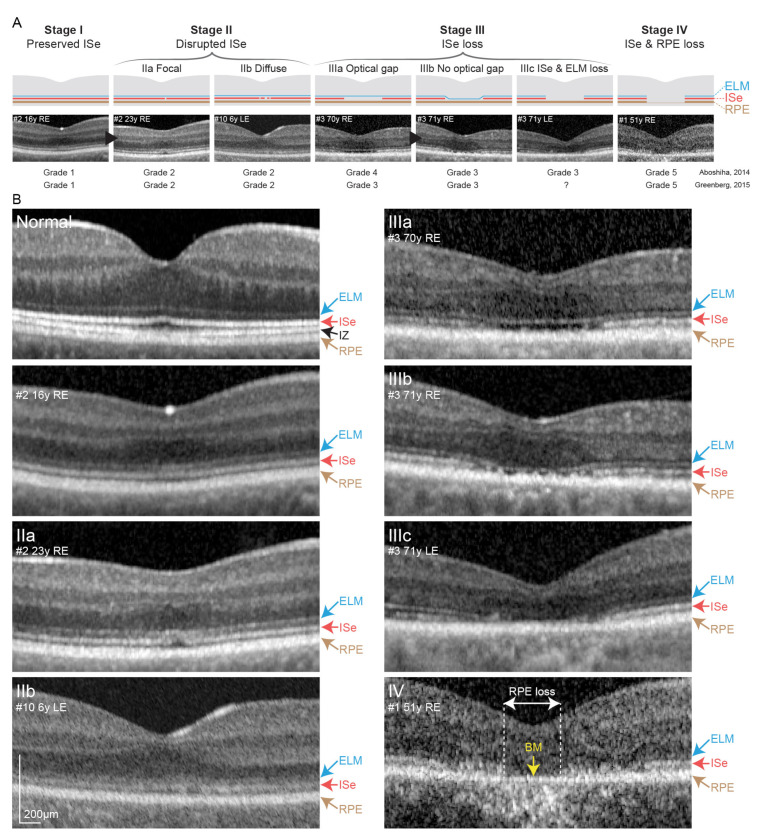
Proposed OCT staging. (**A**) Proposed OCT staging that was created based on observations from Figure 1. Representative OCT figure is shown for each stage. Cases, where progression was observed, are marked with a black arrowhead. Grading based on the previous proposal by Aboshiha et al. [22] and Greenberg et al. [19] is shown below. Note the differences in grades 3 and 4. (**B**) Enlarged OCT images from (**A**). A normal OCT image is placed on the top left, showing the normal foveal structure and the presence of the interdigital zone between the ISe and RPE. Stages IIIa-c are characterized by ISe loss but differ in the presence/absence or shape of ELM. Stage IV is characterized by RPE loss (marked with white arrows). Bruch’s membrane remains as a thin hyperreflective line in that area (marked with yellow arrow), while increased reflectivity of the choroid may be seen. Abbreviation explanation: RE—right eye, LE—left eye, ELM—external limiting membrane, Ise—inner segment elipsoid, IZ—interdigitation zone, RPE—retinal pigment epithelium, BM—Bruch’s membrane.

Due to retrograde change in OCT grade (4→3), according to Aboshiha et al. in patient #3, a modified OCT categorization was proposed. It comprises four stages: (I) preserved ISe, (II) disrupted ISe, (III) ISe loss, and (IV) ISe and retinal pigment epithelium (RPE) loss, with subcategories shown in Figure 4. Previous grades of 3 and 4 (both representing ISe loss with preserved RPE) were joined in stage III. The correlation using this staging was stronger (r = 0.79; *p* < 0.01).

#### 3.2.3. Electroretinography

All patients underwent full-field electroretinography. The four patients younger than 14 months were tested using the Great Ormond Street Hospital (GOSH) protocol [35], while the remaining patients were tested according to the International Society for Clinical Electrophysiology of Vision (ISCEV) standard (Table 2). The rod responses were reduced in three cases (one 7-month-old child and two adults). The mean b-wave amplitude of dark-adapted 0.01 ERG was reduced to 33%. The cone responses were reduced below the noise level in all patients. As the standard light-adapted ERGs are normally dominated by long- and medium-wavelength sensitive cones, short-wavelength (S-cones) with their smaller amplitude and slower kinetics are not visible in the standard protocol. Therefore, extended ISCEV protocol [37] with blue pulses on a long-wavelength selective background was used in two cases, and the ERG remained undetectable. Three patients were measured first as infants, and later on, as older children, the time between both tests ranged from 4.5 to 12 years. The longitudinal analysis showed no progression.

### 3.3. Correlation between Oct Stage and Age from Published Data

**Figure 5:**
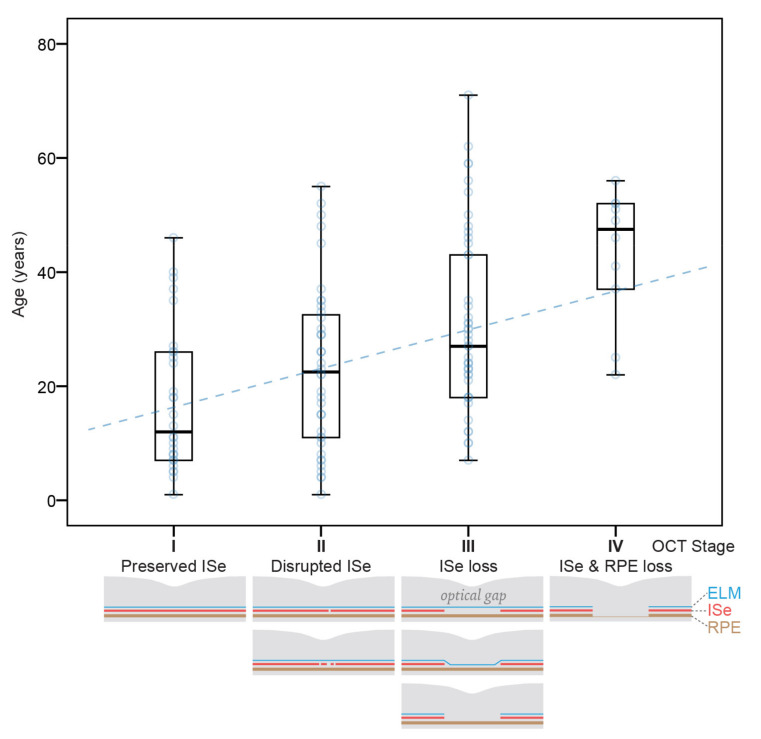
Distribution of patients’ age in different OCT stages. Boxplot chart showing age distribution in groups of patients with OCT stages I-IV. Data from 126 patients were gathered from 7 studies, including ours (Table S1). Possible structural features present in different stages are shown with diagrams at the bottom. Note the increasing age of patients with increasing OCT stage. Horizontal lines represent the median values, boxes represent half of the data for each stage, and whiskers represent the remaining data. Data points for each case are shown with blue circles, and the dashed blue line shows the slope of the regression line.

**Figure 6:**
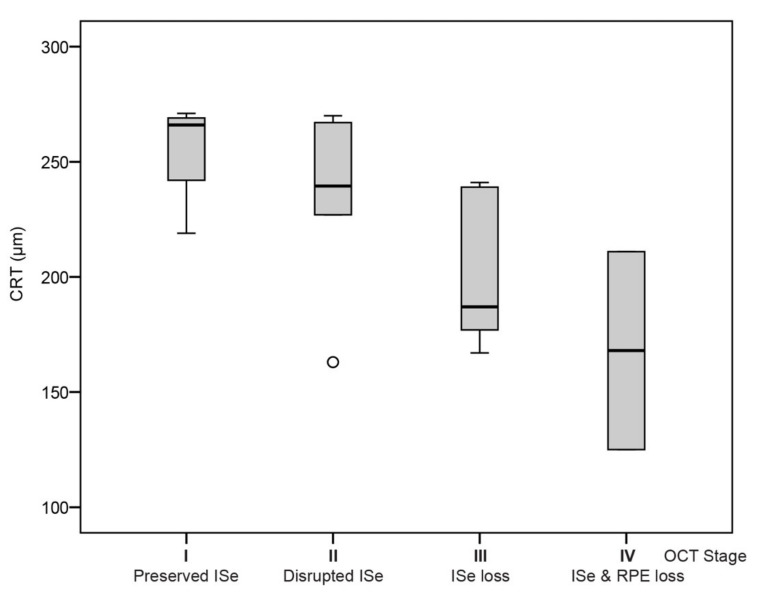
Boxplot chart showing central retinal thickness associated with different OCT stages. Central retinal thickness in patients with OCT stages I-IV. Data were gathered from our study and Brunetti et al. (Table S1). Note the significant decrease in CRT in Stages III and IV, where ISe loss occurs. Horizontal lines represent the median values, boxes represent half of the data for each stage, and whiskers represent the remaining data except in the case of the outlers (circle).

Data from this and six other studies [14,17,19,20,25,36] providing OCT gradings or images of *CNGA3* and *CNGB3* patients were joined with data from the present study in order to inspect the possible correlation of OCT stage with age on a large cohort. In total, there were 126 patients aged 1–71 years (Table S1). Multiple regression analysis showed a significant correlation between age and the four disease stages (*p* < 0.001), with no correlation with genotype (*p* > 0.05). The median ages of patients with stages I–IV were 12 years (*n* = 30), 23 years (*n* = 40), 27 (*n* = 46) and 48 years (*n* = 10), respectively (Figure 5 and Figure 6). CRT measured with Spectralis was also ascertained from our study and Brunetti et al. (total of 17 patients). Median CRT of patients with stages I–IV was 266 μm (*n* = 4), 240 μm (*n* = 6), 187 μm (*n* = 5) and 168 μm (*n* = 2). Multiple regression analysis showed significant correlation between CRT and disease stage (*p* < 0.01) but not gene (*p* > 0.05).

## 4. Discussion

The present study presents the Slovenian *CNGA3/CNGB3* cohort of 12 patients who harbored mostly previously described pathogenic variants and presented with typical clinical features. A simplified OCT staging of changes in the macula was developed based on longitudinal observations. In the second part, a cross-sectional analysis of 126 patients from seven studies, including ours, was performed, which revealed an age-related progression of OCT stages.

### 4.1. Genetic Findings in the Slovenian Cohort

Among the ACHM-related genes, only variants in *CNGA3* and *CNGB3* genes were found in the Slovenian IRD patient cohort, which corroborates the previous estimation that these two genes explain more than 70% of cases with ACHM [38]. We detected several recurrent pathogenic variants, previously reported in patients with ACHM, either in homozygous or compound heterozygous state. The *CNGB3*:p.Thr383IlefsTer13 that was found in three Slovenian patients (four alleles) has been previously shown to have resulted from a founder effect and accounts for over 70–75% of all *CNGB3* alleles [5,39]. In addition, a novel variant c.2104-2A>G in the *CNGB3* gene was identified, which affects the canonical splicing site and presumably results in a loss-of-function, a previously described mechanism of *CNGB3*-retinopathy [38]. In one patient, a pathogenic in-frame duplication of exon 7 in the *CNGB3* was identified using whole-genome sequencing, while the other pathogenic variant was detected by exome sequencing. A comprehensive approach using whole-exome sequencing as the first-tier analysis, followed by targeted Sanger sequencing for family segregation studies and whole-genome sequencing, successfully identified the underlying causative genetic variant in this cohort.

### 4.2. Visual Function

There was no significant deterioration of visual acuity with age, which is consistent with some previous studies [1], but not others [20]. Interestingly, an increase in visual acuity was observed in the first decade. This could be related to visual development, which occurs mainly in the first five years [40] but also a more accurate measurement of VA in older patients. As the daytime vision of patients with ACHM is presumably governed by rods [17], cone degeneration would not be expected to be directly associated with visual acuity decline.

### 4.3. Electroretinography

Electrophysiological findings showed congenital cone dysfunction, as cone-mediated responses were undetectable in all patients tested in their infancy, using GOSH protocol, as well as in older patients, using ISCEV protocol. A similar dysfunction was described in a previous study of ERG characteristics in infants with infantile nystagmus syndrome in a baby in whom ACHM was later confirmed [41]. A longitudinal analysis on three patients who were measured first as infants and later as older children did not show a progression of cone dysfunction. Although the genetic defect is cone-specific in ACHM, anomalies in the rod pathway signaling and defects in postreceptoral responses have been reported [16]. In our study, three out of 12 patients had reduced rod function (one 9-month child with gene *CNGA3* and two adults aged 20 and 70 years with gene *CNGB3* and *CNGA3*, respectively). In a similar study, rod responses were reduced in 59% of patients [42]. However, in the study of Zobor (2017), using ISCEV dark-adapted 0.01 ERG, rod response amplitudes and implicit times were within normal limits [17]. In terms of visual electrodagnostics, our study shows a stationary disease with absent cone responses and, in some patients, abnormal rod responses already early in infancy, showing no progression over time. No improvement of cone function on full-filed ERG is therefore expected after gene therapy [17]. Because most structural abnormalities are observed in the macula, using OCT and AF, it would be interesting to record multifocal ERG to access macular function. Unfortunately, it is hard to get reproducible mfERG responses in ACHM patients due to common nystagmus and photophobia.

### 4.4. CNGA3/CNGB3 Retinopathy Is Predominantly a Progressive Disease

Data gathered from seven studies, including ours, formed the largest *CNGA3/CNGB3* cohort to date (126 patients) with a large age range (1–71 years) [14,17,19,20,25,36]. The cross-sectional analysis showed evidence that *CNGA3/CNGB3* retinopathy is a progressive disease that follows four OCT stages; (I) preserved ISe, (II) disrupted ISe, (III) ISe loss, and (IV) ISe, and RPE loss, which occur slowly over decades.

ACHM has previously been considered a predominately stable condition [25], and although suggested by some authors, predominant structural degeneration has not been confirmed definitively in a large cohort. One cross-sectional study found the presence of hyperreflective zone on OCT age-dependent [43], and another reported that patients presenting with higher OCT grades were significantly older [20]; however, others considered the influence of age and/or follow-up time on change in foveal morphology on OCT unlikely [17,19,25]. These cross-sectional studies were disadvantaged due to a low number of patients and a limited age range. Longitudinal studies have also been performed to inspect possible progression; however, mostly with a very short follow-up, which is likely the reason why the progression was observed in the minority of the patients. Aboshiha, for example, reported progressive outer retinal OCT changes in only 2/38 (5%) patients with a mean follow up time of 20 months [22], Hirji et al. described progressive outer retinal changes in 6/50 (12%) patients, with a mean follow-up time of 5 years [25]. Recently Brunetti-Pierri observed progression of outer foveal structural changes in only 2/21 (12.5%) patients with a mean follow-up of more than 5 years [20]. This was also the case in the Slovenian cohort, where only 3/8 patients showed progression during follow-up of 1–9 years. Thomas et al. found mild changes in Ise in all (5) children, with a mean follow-up of 13 months, but not in 3 adults [28].

In their longitudinal study, Brunetti-Pierri et al. recently observed a decrease in CRT with age, which is concordant with the results of our analysis. However, unlike our results, the authors also observed a functional decline over the years, with deteriorating BCVA and macular sensitivity [20]. If the functional visual loss in ACHM does occur, it remains challenging to observe due to its slow progression.

Animal models support the proposed degenerative nature of *CNGA3/CNGB3* retinopathy. Progressive loss of photoreceptors has been demonstrated in murine models of the *CNGA3* and *CNGB3*-associated ACHM [44,45], while a canine model of *CNGB3*-associated ACHM showed slow progression of cone loss with many cones remaining functional in older dogs [46]. An age-dependent complete restoration of visual acuity in *CNBGB3^-/-^* mice following gene therapy has been observed, with complete visual acuity restoration possible only in younger mice [47]. A similar pattern of age-dependent restoration of vision was observed after gene therapy in the *CNGB3*- canine model of ACHM [46].

A large overlap in age distribution was present in the four stages, which suggests variability in disease. It is possible that *CNGA3* and *CNGB3* cause a spectrum of disorders between stationary progressive disease with different rates of progression, where different variants or other *cis/trans* factors affect disease expression. For example, *RHO* variants may cause stationary (congenital stationary night blindness) or progressive disease (retinitis pigmentosa) even within the same family [48]. It remains to be answered whether the same is true for *CNGA3/CNGB3*.

It would also be important to determine whether the loss of cone photoreceptors in the macula is accompanied by the loss of photoreceptors in the peripheral retina. If those remained functional, gene therapy could still bring benefit to treated patients as they could allow color vision in the peripheral visual field.

According to our cross-sectional analysis, the OCT stages occur over several decades of life; therefore, a longitudinal study of >20 years would be needed to reveal whether morphological changes in time follow the proposed four-stage linear pattern.

### 4.5. Comparison between CNGA3 and CNGB3 Patients

There were no statistically significant differences in VA or OCT stages between *CNGA3* and *CNGB3* patients in the Slovenian cohort when multiple regression analysis accounting for age was performed. There were also no notable differences in color vision, foveal hypoplasia, FAF patterns or electrophysiology, although the data were insufficient for formal statistical analysis.

Multiple regression analysis on the 126-patient cohort gathered from seven studies also showed no significant correlation between OCT stage and gene while confirming age-related structural progression.

### 4.6. Study Limitation and Strengths

The limitation of the study is the lack of longer longitudinal data that could show a linear progression of structural changes in individual patients. Another limitation is a lack of rigorous follow-up patient data. OCT and FAF were able to show only structural changes in the macula only, but not in the peripheral retina. Moreover, OCT and FAF imaging data were limited on the follow-up and were not available in all patients. The oldest patient at 71 years of age showed some FAF abnormalities in the peripheral retina. A wide-field FAF would probably give additional information in other older patients. ERG is unfortunately not very useful in detecting the structural progression of cones, as their responses are usually absent from the start. Another limitation is the lack of variant-specific analysis. Although there was no difference detected between the *CNGA3* and *CNGB3* patients, some variability could still exist due to the different effects of specific variants within the two genes. The strengths of the study include relatively long follow-up of the Slovenian cohort and a wide age range of patients (up to 71 years). However, the main strength of the study is a cross-sectional approach on pooled data from seven patient cohorts.

## 5. Conclusions

A cross-sectional analysis of the largest patient cohort to date suggests that biallelic variants in *CNGA3* and *CNGB3* in most patients result in slow retinal degeneration that occurs over decades of life. Whether the same is true for the visual function remains to be confirmed. Proposed OCT staging of changes in the macula follows the patterns of (I) preserved Ise, (II) disrupted ISe, (III) ISe loss and (IV) ISe and RPE loss. Structural degeneration is especially important to acknowledge when considering genetic modification of dysfunctional cones.

## Figures and Tables

**Table 1:** Clinical characteristics and genetic findings of the Slovene patients.

Variant	ClinVar ID and Intrepretation	LOVD	GnomAD Allele Frequency	Reference PMID	Functional Study PMID
NM_001298.2(CNGA3):c.829C>T	9481	CNGA3_000033	0,0000955	11536077	20238023
(p.Arg277Cys)	Pathogenic/Likely pathogenic		(no homozygotes)		
NM_001298.2(CNGA3):c.847C>T	9474	CNGA3_000034	0,0000995	9662398, 26992781, 24504161, 11536077, 25637600, 9662398, 25637600	17693388, 20238023
(Arg283Trp)	Pathogenic/Likely pathogenic		(no homozygotes)		
NM_001298.2(CNGA3):c.1279C>T	497256	CNGA3_000038	0,000395	11536077, 23972307, 28559085, 18445228	18445228
(Arg427Cys)	Pathogenic		(1 homozygote)		
NM_001298.2(CNGA3):c.1641C>A	9478	CNGA3_000044	0,000151	14757870, 23972307, 30682209, 11536077, 31456290, 9662398	17693388
(p.Phe547Leu)	Pathogenic/Likely pathogenic		(1 homozygote)		
NM_019098.4(CNGB3):c.1578+1G>A	189031	CNGB3_000034	0,0000199	10958649, 12187429	n/a
	Pathogenic/Likely pathogenic		(no homozygotes)		
NM_019098.4(CNGB3):c.1148delC (p.Thr383IlefsTer13)	5225	CNGB3_000001	0,00172	25770143, 17265047	12815043, 23805033
	Conflicting interpretations of pathogenicity		(2 homozygotes)		
NM_019098.4(CNGB3):c.2104-2A>G	Novel variant (PVS1, PM2); Likely pathogenic	n/a	n/a	15657609	n/a
NM_019098.4(CNGB3):c.819_826delCAGACTCC (p.Arg274ValfsTer13)	374027	CNGB3_000044	0,0000517	10888875, 20079539, 29769798	n/a
	Pathogenic		(no homozygotes)		
CNGB3:dup ex7	Pathogenic/Likely pathogenic	n/a	n/a	28795510	n/a
(NC_000008.11:g.86,652,314_86,662,912dup)					

Abbreviation explanation: LOVD—Leiden open-source validation database, GnomAD—Genome aggregation database, PMID—Pubmed ID.

**Table 2:** Spectrum of the identified *CNGA3* and *CNGB3* variants in the studied Slovene cohort.

ID	Sex	Gene	Variants	Nysta-Gmus	Foveal Hypo-Plasia	Age at Exam	Color Vision (Ishihara)	BCVA	BCVA Better Eye Decimal (logMAR)	OCT Machine	CRT OD (um)	CRT OS (um)	OCT Grade	Foveal FAF (BE)	ERG Rod Response	ERG Cone Response	Measuring Standard
1	M	CNGA3	p.Arg277Cys (hmz)	no	1a	51	n/a	RE 0.16 LE 0.16	0.16 (0.80)	Spectralis	211	233	BE 5	decreased with hyperAF ring	normal	undetectable	ISCEV
2	F	CNGA3	p.Arg283Trp (hmz)	yes	1b	4	n/a	RE 3/16 LE 2/16	0.19 (0.73)	n/a	n/a	n/a	n/a	n/a	normal	degraded	GOSH
						16	RE 0/15 LE 0/15	RE 0.1 LE 0.1	0.1 (1.00)	Spectralis	268	271	BE 1	decreased	normal	undetectable	ISCEV
						23	RE 1/15 LE 1/15	RE 0.2 LE 0.2	0.2 (0.70)	Spectralis	267	269	RE 2 LE 1	n/a	n/a	n/a	n/a
3	F	CNGA3	p.Arg283Trp (hmz)	yes	1a	70	n/a	RE CF 0.5 m LE 0.1	0.1 (1.00)	Spectralis	180	224	RE 3 LE 4	decreased with hyperAF ring	reduced	undetectable	ISCEV extended
						71	RE 0/15 LE 1/15	RE CF 0.5 m LE CF 2 m	0.03 (1.52)	Spectralis	177	219	RE 3 LE 3	decreased with hyperAF ring	n/a	n/a	n/a
4	F	CNGA3	p.Phe547Leu (hmz)	no	1a	56	n/a	RE 0.1 LE 0.16	0.16 (0.80)	Spectralis	247	242	BE 4	decreased with hyperAF ring	normal	undetectable	ISCEV
						59	RE 1/15 LE 1/15	RE 0.1 LE 0.15	0.15 (0.82)	Spectralis	241	235	BE 4	decreased with hyperAF ring	n/a	n/a	n/a
5 *	M	CNGA3	p. Arg283Trp; p.Phe547Leu	yes	no	9 m	n/a	binocular ≥ 6/190	0.03 (1.52)	n/a	n/a	n/a	n/a	n/a	normal	undetectable	GOSH
						14 m	n/a	n/a	n/a	n/a	n/a	n/a	n/a	n/a	normal	undetectable	ISCEV with skin electrodes
						2	n/a	binocular 6/12 at 0.5 m	0.042 (1.38)	Triton	216	207	BE 1	n/a	n/a	n/a	n/a
						8	n/a	binocular 6/24	0.25 (0.60)	Triton	198	200	BE 1	n/a	n/a	n/a	n/a
6 *	F	CNGA3	p. Arg283Trp; p.Phe547Leu	yes	no	6 m	n/a	binocular ≥ 6/620	0.01 (2.00)	n/a	n/a	n/a	n/a	n/a	n/a	n/a	n/a
						9 m	n/a	n/a	n/a	n/a	n/a	n/a	n/a	n/a	normal	undetectable	GOSH
						2	n/a	binocular ≥ 6/620	0.01 (2.00)	Triton	172	179	BE 1	n/a	n/a	n/a	n/a
						5	n/a	binocular 6/24	0.25 (0.60)	Triton	192	182	BE 1	increased	n/a	n/a	n/a
7	M	CNGA3	Arg265Trp; p.Arg409Cys	yes	no	5 m	n/a	binocular 6/130	0.046 (1.34)	n/a	n/a	n/a	n/a	n/a	n/a	n/a	n/a
						7 m	n/a	n/a	n/a	n/a	n/a	n/a	n/a		reduced	undetectable	GOSH
						4	n/a	binocular 0.1	0.1 (1.00)	Triton	251	195	RE 2 LE 1	n/a	n/a	n/a	n/a
						5	n/a	RE 0.15 LE 0.15	0.15 (0.82)	n/a	n/a	n/a	n/a	n/a	n/a	n/a	n/a
8	F	CNGB3	p.Thr383IlefsTer13 (pat,mat), c.2104-2A>G (pat)	yes	1b	5 m	n/a	n/a	n/a	n/a	n/a	n/a	n/a	n/a	n/a	n/a	n/a
						6	n/a	RE 0.3 LE 0.2	0.3 (0.52)	n/a	n/a	n/a	n/a	n/a	n/a	n/a	n/a
						11	1/12	RE 0.1 LE 0.1	0.1 (1.00)	n/a	n/a	n/a	n/a	n/a	n/a	n/a	n/a
						15	3/12	BE 4/24 (E signs)	0.17 (0.78)	n/a	n/a	n/a	n/a	n/a	n/a	n/a	n/a
						20	n/a	RE 0.1 LE 0.1	0.1 (1.00)	n/a	n/a	n/a	n/a	n/a	reduced	undetectable	ISCEV
						21	n/a	n/a	n/a	n/a	n/a	n/a	n/a	n/a	reduced	undetectable	ISCEV extended
						22	n/a	n/a	n/a	Triton	214	205	BE 4	n/a	n/a	n/a	n/a
						26	n/a	RE 0.05 LE 0.05	0.05 (1.30)	n/a	n/a	n/a	n/a	n/a	n/a	n/a	n/a
						31	RE 3/15 LE 2/15	RE CF 1.5 m LE CF 1.5 m	0.025 (1.60)	Triton	216	205	BE 4	decreased with hyperAF ring	n/a	n/a	n/a
9 ’	M	CNGB3	p.Arg274ValfsTer13; dup ex7	yes	1a	3 m	n/a	RE 6/500 LE 6/500	0.012 (1.92)	n/a	n/a	n/a	n/a	n/a	normal	undetectable	GOSH
						7 m	n/a	n/a	n/a	n/a	n/a	n/a	n/a	n/a	normal	undetectable	GOSH
						2	n/a	binocular 6/18	0.33 (0.48)	n/a	n/a	n/a	n/a	n/a	n/a	n/a	n/a
						6	n/a	RE 0.2 LE 0.4	0.4 (0.40)	Spectralis	274	271	BE 2	decreased	n/a	n/a	n/a
						7	n/a	n/a	n/a	n/a	n/a	n/a	BE 2	decreased	normal	undetectable	ISCEV with skin electrodes
						8	n/a	RE 0.25 LE 0.6	0.6 (0.22)	Spectralis	270	271	BE 2	n/a	n/a	n/a	n/a
10 ’	F	CNGB3	p.Arg274ValfsTer13; dup ex7	yes	1a	4 m	n/a	binocular 6/130	0.046 (1.34)	Triton	n/a	n/a	RE 1 LE n/a	n/a	normal	undetectable	GOSH
						5	n/a	RE 0.1 LE 0.1	0.1 (1.00)	Spectralis	237	245	BE 1	n/a	normal	undetectable	ISCEV with skin electrodes
						6	n/a	RE 0.2 LE 0.2	0.2 (0.70)	Triton	219	206	BE 2	n/a	n/a	n/a	n/a
11 ”	F	CNGB3	c.1578+1G>A; p.Thr383IlefsTer13	yes	n/a	6 m	n/a	binocular 6/620	0.01 (2.00)	n/a	n/a	n/a	n/a	n/a	normal	undetectable	GOSH
						14 m	n/a	n/a	n/a	n/a	n/a	n/a	n/a	n/a	normal	undetectable	GOSH
						2	n/a	binocular 0.1 at 2 m	0.033 (1.48)	n/a	n/a	n/a	n/a	n/a	n/a	n/a	n/a
12 ”	M	CNGB3	c.1578+1G>A; p.Thr383IlefsTer13	yes	n/a	7 m	n/a	binocular 6/1000	0.006 (2.22)	n/a	n/a	n/a	n/a	n/a	n/a	n/a	n/a
						1	n/a	binocular 6/620	0.01 (2.00)	n/a	n/a	n/a	n/a	n/a	normal	undetectable	GOSH

Patients from the same families are marked with *, ’ and ”, respectively. In patients 6, 10 and 12, targeted Sanger sequencing of known familial pathogenic variants was performed. Abbreviation explanation: RE—right eye, LE—left eye, BE—both eyes, BCVA—best-corrected visual acuity, OCT—optical coherence tomography, ERG—electroretinography, ISCEV— International Society for Clinical Electrophysiology of vision protocol, GOSH— Great Ormond Street Hospital protocol.

## Data Availability

The data presented in this study are available on request from the corresponding author. The data are not publicly available due to personal data protection.

## Supplementary Materials

The Supplementary Materials are available online at https://www.mdpi.com/article/10.3390/cimb43020067/s1.

## Abbreviations

ACHM	achromatopsia
BCVA	best corrected visual acuity
BE	both eyes
CNGA3	Cyclic Nucleotide Gated Channel Subunit Alpha 3
CNGB3	Cyclic Nucleotide Gated Channel Subunit Beta 3
CNV	copy number variation
DA	dark-adapted
ELM	external limiting membrane
ERG	electroretinography
FAF	fundus autofluorescence
GOSH	Great Ormond street hospital protocol
ISCE	International Society for Clinical Electrophysiology of vision protocol,
Ise band	inner segment ellipsoid band
LE	left eye
OCT	optical coherence tomography
RE	right eye
RGB	red green blue
RPE	retinal pigment epithelium
UMC LJ	University Medical Centre Ljubljana
VF	perimerty
VEP	visual evoked potentials
