# Supplementary figures and images for: Disease Progression in CNGA3 and CNGB3 Retinopathy; Characteristics of Slovenian Cohort and Proposed OCT Staging Based on Pooled Data from 126 Patients from 7 Studies
Source: Curr Issues Mol Biol. 2021 Aug 16;43(2):941–57. doi: 10.3390/cimb43020067 (PMC8929018; doi:10.3390/cimb43020067)

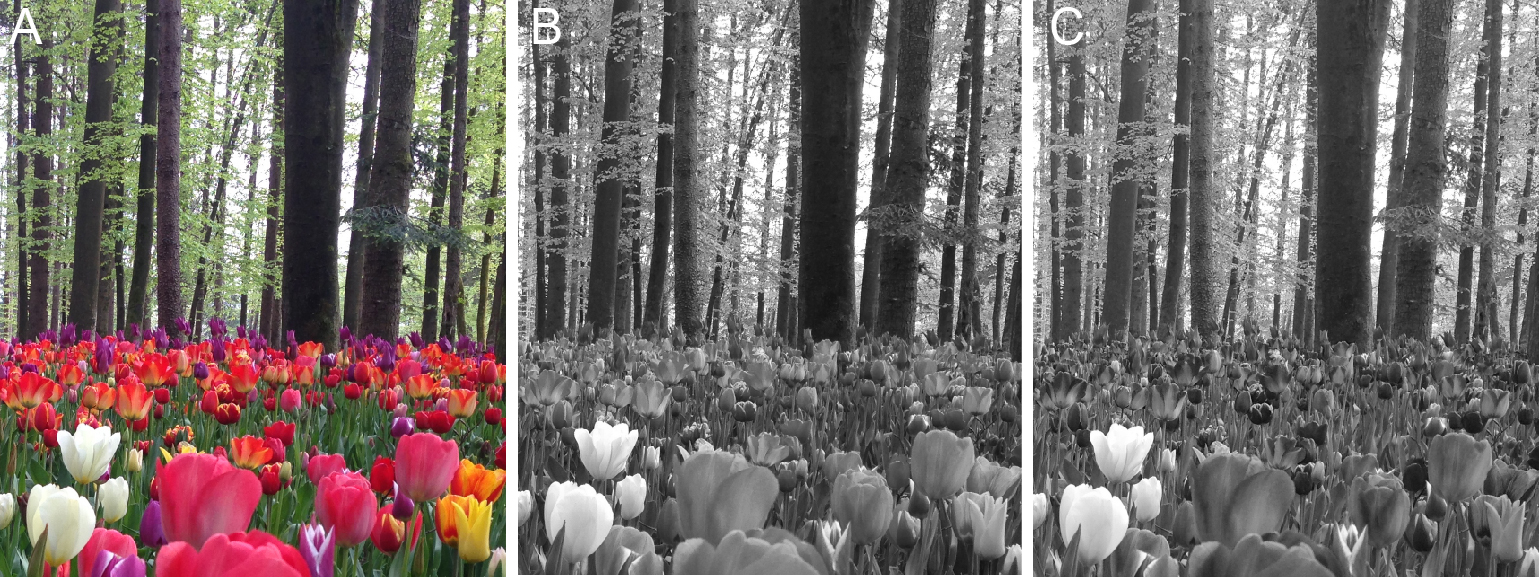

Supplement: Supplementary file 1 [file cimb-43-00067-s001.zip › Figure S1.jpg]
